# Effects of the DICE Method to Improve Timely Recognition and Treatment of Neuropsychiatric Symptoms in Early Alzheimer’s Disease at the Memory Clinic: The BEAT-IT Study

**DOI:** 10.3233/JAD-230116

**Published:** 2023-06-13

**Authors:** Willem S. Eikelboom, Esther van den Berg, Michiel Coesmans, Jeannette A. Goudzwaard, Marc Koopmanschap, Najoua Lazaar, Rozemarijn L. van Bruchem-Visser, Jan J.M. Driesen, Tom den Heijer, Susanne Hoogers, Frank Jan de Jong, Francesco Mattace-Raso, Elsbeth C. Thomeer, Suzanne Vrenken, Lilian J.H.M. Vroegindeweij, Sytse U. Zuidema, Ellen H. Singleton, John C. van Swieten, Rik Ossenkoppele, Janne M. Papma

**Affiliations:** a Department of Neurology and Alzheimer Center Erasmus MC, Erasmus MC, University Medical Center, Rotterdam, The Netherlands; b Department of Psychiatry, Erasmus MC, University Medical Center, Rotterdam, the Netherlands; c Department of Internal Medicine, Erasmus MC, University Medical Center, Rotterdam, the Netherlands; d Erasmus School of Health Policy & Management, Erasmus University, Rotterdam, the Netherlands; e Department of Neurology, Franciscus Vlietland, Schiedam, The Netherlands; f Department of Neurology, Franciscus Gasthuis, Rotterdam, The Netherlands; g Department of Neurology, Spijkenisse Medical Center, Spijkenisse, The Netherlands; h Department of Neurology, Maasstad Hospital, Rotterdam, The Netherlands; i Department of Geriatrics, Spijkenisse Medical Center, Spijkenisse, The Netherlands; j Department of Neurology, Het Van Weel-Bethesda Ziekenhuis, Dirksland, The Netherlands; k Department of General Practice and Elderly Care Medicine, University of Groningen, University Medical Center Groningen, Groningen, The Netherlands; l Department of Neurology, Alzheimer Center Amsterdam, Amsterdam University Medical Center, Amsterdam, The Netherlands; m Clinical Memory Research Unit, Lund University, Malmö, Sweden

**Keywords:** Alzheimer’s disease, apathy, behavioral and psychological symptoms of dementia, delivery of care, dementia, depression, neuropsychiatric inventory, neuropsychiatric symptoms

## Abstract

**Background:**

Neuropsychiatric symptoms (NPS) are highly prevalent in Alzheimer’s disease (AD) and are associated with negative outcomes. However, NPS are currently underrecognized at the memory clinic and non-pharmacological interventions are scarcely implemented.

**Objective:**

To evaluate the effectiveness of the Describe, Investigate, Create, Evaluate (DICE) method™ to improve the care for NPS in AD at the memory clinic.

**Methods:**

We enrolled sixty community-dwelling people with mild cognitive impairment or AD dementia and NPS across six Dutch memory clinics with their caregivers. The first wave underwent care as usual (*n* = 36) and the second wave underwent the DICE method (*n* = 24). Outcomes were quality of life (QoL), caregiver burden, NPS severity, NPS-related distress, competence managing NPS, and psychotropic drug use. Reliable change index was calculated to identify responders to the intervention. A cost-effectiveness analysis was performed and semi-structured interviews with a subsample of the intervention group (*n* = 12).

**Results:**

The DICE method did not improve any outcomes over time compared to care as usual. Half of the participants of the intervention group (52%) were identified as responders and showed more NPS and NPS-related distress at baseline compared to non-responders. Interviews revealed substantial heterogeneity among participants regarding NPS-related distress, caregiver burden, and availability of social support. The intervention did not lead to significant gains in quality-adjusted life years and well-being years nor clear savings in health care and societal costs.

**Conclusion:**

The DICE method showed no benefits at group-level, but individuals with high levels of NPS and NPS-related distress may benefit from this intervention.

## INTRODUCTION

Neuropsychiatric symptoms (NPS) are highly prevalent in the early clinical stages of Alzheimer’s disease (AD) [1, 2]. These symptoms are related to negative clinical outcomes such as accelerated disease progression [3], lowered quality of life (QoL) [4], increased caregiver burden [5], and earlier nursing home placement [6]. NPS are also associated with increased formal healthcare utilization and informal care leading to major healthcare costs [7, 8]. The clinical importance of NPS in early clinical stages of AD is further highlighted by the concept of mild behavioral impairment (MBI) that describes individuals with late-life onset of persistent NPS in the context of no or mild cognitive impairment (MCI) [9]. Recent studies have shown that individuals with MBI have an elevated risk for progression to dementia[10, 11].

The etiology of NPS in AD is multifactorial and consists of potential modifiable psychosocial causes such as unmet needs, negative communication style of caregivers, and environmental stressors [12, 13]. Therefore, international guidelines recommend non-pharmacological interventions as first-line-treatment for NPS [14–16]. Previous systematic reviews have shown the effectiveness of various non-pharmacological approaches in reducing NPS severity, NPS-related distress among caregivers, and psychotropic drug use in AD populations [17–22]. Moreover, investing in non-pharmacological interventions for NPS is shown to be cost-effective [23]. For community-dwelling individuals with early-stage AD, most promising approaches include the Tailored Activity Program to increase tailored meaningful activities [24, 25] and psychoeducation programs that enhance knowledge about AD and underlying causes of NPS in AD and provide caregivers with new skills to manage NPS[26–28].

Given the clinical relevance of NPS and the availability of evidence-based interventions, timely detection and treatment of NPS has potential clinical benefits for people with AD dementia and their caregivers [29, 30]. The memory clinic may be an ideal setting for early assessment and management of NPS in early AD dementia, as these multidisciplinary facilities offer a comprehensive diagnostic work-up and have the potential to timely detect NPS and to offer post-diagnostic care [31]. However, NPS are currently underdiagnosed and non-pharmacological interventions are hardly implemented in individuals who visit the memory clinic with early AD dementia [32, 33]. Instead, NPS are often considered as medication targets [34], leading to high rates of off-label prescription of psychotropic drugs that are at best only modestly effective in dementia and are associated with serious side effects [35, 36].

Hence, there is a need for a tool that translates the current international guidelines into clinical practice and integrates a comprehensive assessment of NPS into the standard work-up at memory clinics in order to improve early recognition and tailored treatment of NPS in AD. The Describe, Investigate, Create, Evaluate (DICE) method™ provides such a tool [37]. This person-centered framework uses a step-by-step approach to describe NPS in the context in which they occur, investigate possible underlying causes and triggers, create interventions targeting the underlying causes and triggers that have been identified, and subsequently evaluate the effectiveness and implementation of these interventions. Recent studies have evaluated the effectiveness of the DICE method in caregivers and care professionals of individuals with dementia living at home [38–40]. These studies showed that a one-day training on the application of the DICE method increased knowledge about NPS and improved confidence in managing NPS among both family caregivers and care professionals using a pre-post design [39, 40]. In addition, a pilot randomized controlled trial showed that a web-based tool based on the DICE approach reduced NPS-related distress among caregivers of individuals with dementia [38]. Although the DICE method has been suggested as the most promising non-pharmacological intervention to diagnose and treat NPS in dementia [41], no studies have been conducted that have evaluated the use of this method in people with AD dementia in the memory clinic setting.

The aim of the current intervention study was to evaluate the effectiveness of the DICE method used to structure and standardize the care for NPS in early AD at the memory clinic as part of the BEhavioral symptoms in Alzheimer’s disease Towards early Identification and Treatment (BEAT-IT) study [42]. We hypothesized that improving early assessment and adequate management of NPS would improve the QoL of patients with early AD dementia and their caregivers.

## METHODS

This trial was registered on the Netherlands Trial Registry (NTR7459). Detailed information on the design and the intervention components was described prior to starting the intervention [42]. This study was conducted and reported following the CONSORT guideline (Supplementary Table 1).

### Study design

This was a multicenter study with a quasi-experimental design. Participants were recruited from the following six memory clinics located in the greater Rotterdam area, the Netherlands: Erasmus MC University Medical Center, Franciscus Gasthuis, Franciscus Vlietland, Het Van Weel-Bethesda Ziekenhuis, Maasstad Hospital, and Spijkenisse Medical Center. Patients were enrolled together with their primary caregiver in two waves. The first wave of participants was offered care as usual at their local hospital and served as a control group. As the enrollment of the first wave was completed, a second wave of participants was recruited at the same hospitals, and all underwent the DICE method at the Erasmus MC University Medical Center.

### Participants

Participants were eligible to participate if they met all of the following criteria: (a) a clinical diagnosis of MCI with AD as the primary suspected etiology [43], AD dementia [44], or suspected mixed AD dementia/vascular dementia (VaD) that was established in the memory clinic within the last two years and was based on a neuropsychological assessment and neuroimaging; (b) the presence of NPS as indicated by the Neuropsychiatric Inventory-Questionnaire (NPI-Q) total score ≥1 [45]; (c) a Mini-Mental State Examination (MMSE) score >15 at baseline; (d) patients had to be community-dwelling; and (e) a reliable informal caregiver needed to be available who was considered the primary caregiver. Patients were excluded if they (a) met the criteria of any non-AD neurodegenerative disease, except vascular co-pathology; (b) were legally incapable; (c) showed evidence of current delirium or previous delirium in the past six months; (d) were diagnosed with a primary (premorbid) psychiatric disorder such as schizophrenia or bipolar disorder that could better explain the manifestation of NPS, or current abuse of alcohol or drugs; or (e) were participating in a clinical trial.

### Procedure

For both waves, potential participants were informed by their attending physician at their local hospital. When both patient and caregiver agreed to participate, they were contacted by a researcher for additional information and screening of the eligibility criteria. We registered reasons for declining participations and monitored reasons for drop-out.

This study was conducted during the COVID-19 pandemic, which affected the enrollment of participants. During the first lockdown in the Netherlands (March 2020–July 2020), we had to stop the recruitment of participants in the control group earlier than planned. Follow-up assessments of participants who were already enrolled were conducted via telephone and questionnaires were send through mail and discussed via telephone. We started the enrollment of the second wave of participants three months after COVID-19 restrictions ended as it took time until the care at the memory clinics normalized, and also in order to minimize the effects of COVID-19 restrictions on study outcomes.

### Control group

Participants in the control group received care as usual at their local hospital. We recorded the care received including clinical follow-up visits, prescription of psychotropic drugs including antidepressants, sedative-hypnotics, antipsychotics or mood stabilizers, prescription of cognitive enhancers including cholinesterase inhibitors or memantine, and referral to case management, mental healthcare, or day care centers.

At baseline, 13 participants (38%) in the control group had a case manager, while eight (24%) were on the waiting list. Furthermore, two participants (6%) went to an adult day care center, while two participants (6%) were on the waiting list. Six participants (17%) used psychotropic medications, and 19 participants (48%) received cognitive enhancers at baseline.

During the six-month study period, 20 participants (59%) visited their local memory clinic for a clinical follow-up visit. No participants were referred to a psychiatrist working at the local memory clinic, while two participants (6%) were referred to external mental healthcare. During the study period, four participants (12%) were referred to a case manager and four participants (12%) were referred to adult day care. Local physicians prescribed new psychotropic medications for two participants (6%) during the six-month study period.

### Intervention group

**Fig. 1:**
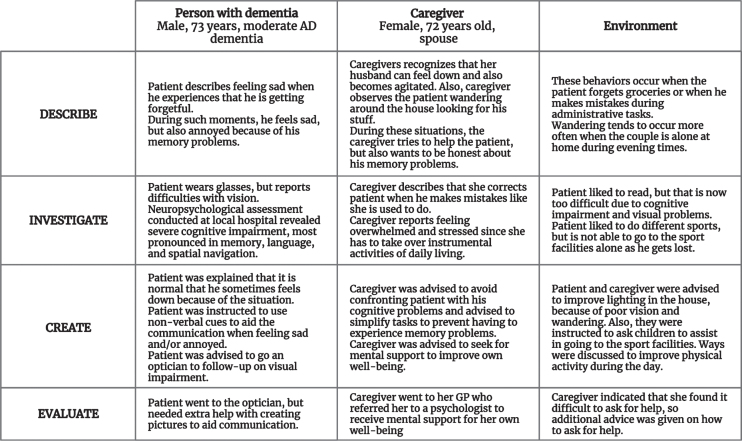
Anonymized case illustrating the Describe, Investigate, Create, Evaluate (DICE) method™.

All participants included in the second wave underwent the DICE method to structure and standardize the assessment and management of NPS in addition to the care as usual received at their local hospital [26]. In short, participants were invited for a first visit, in which NPS were *described* and possible causes of NPS related to the patient, caregiver, and their environment were *investigated*. Thereafter, participants were discussed during a multidisciplinary meeting consisting of neuropsychologists, a psychiatrist, and a geriatrician to *create* a treatment plan based on current guidelines on the diagnosis and treatment of NPS in dementia. During a second visit, the treatment plan was discussed with the participants and adjusted to their wishes. Next, participants were provided with advice on how to manage NPS with a focus on psychoeducation, caregiver support, and increasing meaningful activities. After one month, implementation of strategies was *evaluated* by telephone and adjusted if needed. The intervention itself was carried out by a neuropsychologist (W.S.E.) together with either a psychiatrist (M.C.) or a licensed clinical neuropsychologist (E.v.d.B.). Figure 1 illustrates the use of the DICE method for one participant in which personal details were adjusted to ensure anonymization.

The interventions were delivered as planned for all but two participants. For these two participants, the second visit was replaced by a telephone call with the caregiver as in one participant no NPS were identified after thorough assessment, and in the other participant, the first visit resulted in too much distress for the patient that it was decided to perform the *create* with the partner only.

### Outcome measures

Participants underwent a baseline assessment, a follow-up assessment after three months, and a follow-up assessment after six months. Visits took place at the patients’ home or at the local hospital. As a consequence of the lockdowns during the COVID-19 pandemic, a part of the assessments were conducted via telephone.

### Primary outcomes

Change in QoL measures after three months follow-up were primary outcomes, while we also studied whether effects maintained after six months. QoL was selected as primary outcome as NPS have a substantial impact on the QoL of patients with AD and their caregiver [4, 5]. Although the DICE approach targets NPS in dementia, the intervention may not necessarily directly reduce NPS but rather equip patients and caregivers with betters skills to manage NPS improving their QoL.

QoL of the patient was measured using the Quality of Life in Alzheimer’s Disease (QoL-AD) questionnaire [46]. Patients were questioned via an interview format (score range 13–52), while the proxy version was filled out by the caregiver (score range 13–52). The CarerQol-7D was used to assess care-related QoL in caregivers [47]. The CarerQol-7D includes six burden dimensions and a subjective visual analog scale (VAS) for happiness (score range 0–10). The scores on the six burden dimensions were transformed into a utility score (score range 0–100) by adding up the relative utility weights for each item derived from the Dutch population [48].

### Secondary outcomes

The perseverance time question was used to measure caregiver burden [49]. Caregivers were asked to indicate the time they felt able to maintain care under a hypothetical stable situation using the following categories. We combined the lower four categories (<1 week, 1 week– 1 month, 1–6 months, 6 months– 1 year) as only very few caregivers endorsed these categories. This resulted in three categories: <1 year, 1–2 years, >2 years.

The presence and severity of NPS were assessed using the Dutch NPI-Q [45]. An additional item was added for which caregivers had to rate how confident they feel in managing this symptom (score range 0 = not confident to 4 = extremely confident) [50]. The Behavioral Pathology in Alzheimer’s Disease Rating Scale (BEHAVE-AD) was administered to the caregiver to assesses NPS that are observed in AD [51].

In addition to global measures of NPS, scales for specific NPS were administered when these NPS were endorsed on the NPI-Q at baseline. Although administered in both groups, this procedure was followed to gather information about the manifestation of NPS for the *Describe* step of the intervention. These instruments consisted of the Dutch version of the Cornell Scale for Depression in Dementia (CSDD) and the Rating Anxiety in Dementia (RAID) scale for depressive symptoms and anxiety [52, 53], the Dutch version of the informant-reported Apathy Evaluation Scale (AES-I) for apathy [54], the combined subscales A and B of the BEHAVE-AD for delusions and hallucinations [51], the Dutch version of the Cohen-Mansfield Agitation Inventory (CMAI-D) for agitation, irritability, and aberrant motor behavior [55], and the Sleep Disorder Inventory (SDI) for sleep disturbances [56].

Psychotropic medication use was documented during each assessment and was classified as follows: antidepressants, sedative-hypnotics, antipsychotics, or mood stabilizers. In addition, cognitive enhancers such as cholinesterase inhibitors or memantine were also documented [57].

At baseline and after six months follow-up, the Clinical Dementia Rating Scale (CDR) [58] and the MMSE [59] were administered to measure disease severity and global cognitive functioning respectively.

### Qualitative outcomes

All participants of the intervention group were invited to participate in a semi-structured interview after completing the study. These interviews were conducted face-to-face by a researcher (N.L.) who was not involved in the assessments or intervention. All interviews were audio-taped after obtaining verbal informed consent. Topics included NPS-related self-efficacy, knowledge about NPS in dementia, caregiver burden, and experiences with the DICE method. Topics were discussed from both the perspective of patients and caregivers.

### Cost-effectiveness analysis

Patients completed the EQ-5D-5-L to measure health-related QoL [60], and the ICEpop CAPability measure for Older people (ICECAP-O) to assess well-being [61]. In addition, the Institute for Medical Technology Assessment Valuation of Informal Care Questionnaire (iMTA iVICQ) was used to establish the amount, costs, and appraisal of informal care provided by the caregiver who participated in the study [62]. Also, the iMTA Medical Consumption Questionnaire (iMTA MCQ) was administered to assess the healthcare use of the patient in the past three months [63].

### Statistical analysis

Differences in demographic variables and baseline clinical characteristics between the two groups were examined using analysis of variance, Mann-Whitney U tests, or *χ*^2^ tests where appropriate.

#### Quantitative outcomes

We used linear mixed models (LMM) including random intercepts for participant and hospital to investigate differences between the two groups in the outcomes over time. Interaction between group and time after three months and six months were examined, with three months follow-up as primary endpoint. All LMMs were corrected for age of the patient, sex of the patient, and disease stage (MCI/dementia). We selected linear models for all analyses based on the Akaike information criterion and likelihood ratio *χ*^2^ tests. For all LMMs, assumptions were checked by visual inspection of scatterplots of standardized residuals and Q-Q plots. For CarerQol-7D VAS scores, NPI-Q total scores, NPI-Q competence scores, CMAI total scores, SDI total scores, normality slightly deviated. Subsequent sensitivity analyses using bootstrap procedure with 200 bootstrap samples to calculate confidence intervals did not change ourfindings.

To study individual effects of the intervention on the primary outcomes, reliable change index (RCI) was calculated for each participant in the intervention group. The RCI can be used to establish whether a delta score (post-test – pre-test) of an individual participant is statistically significant taking measurement error, test-retest reliability, and treatment-nonspecific changes in the control group into account [64]. The RCI was calculated for the self-reported QoL-AD total score, proxy rated QoL-AD total score, CarerQol-7D utility score, and the CarerQol-7D VAS score across all time points. In addition, we conducted sensitivity analyses using a regression-based approach to take regression to the mean into account. Participants in the intervention group who showed a reliable change (RCI or residual score >1.645) on any of the primary outcomes after three months and/or six months follow-up were referred to as ‘responders’.

LMMs on primary and secondary outcomes were corrected for multiple testing using the Benjamini-Hochberg adjusted false discovery rate (FDR) of 0.05. RCI analyses were considered exploratory and were therefore uncorrected for multiple testing. Analyses were conducted using SPSS version 26.0 and *R* version 4.0 (*lme4*, *splines*, *lmerTest*, and *boot* packages).

#### Qualitative outcomes

The interviews were analyzed using a thematic analysis approach by two independent researchers (W.S.E., N.L.) [65]. These researchers independently proposed a code book consisting of open codes that emerged from the data. Next, these codes were discussed resulting in a final code book, and two researchers systematically coded the data using a combination of open coding, axial coding, and selective coding. Codes were collided into preliminary categories and themes that were redefined following consensus among researchers.

#### Cost-effectiveness

For each patient, the number of quality-adjusted life years (QALYs) and well-being years during the six-month follow-up was calculated as an area under the curve, taking account of the values of the three measurements. To prevent bias in estimates of QALYs and wellbeing years, we adjusted for small imbalances between the groups in baseline values using Manca’s regression-based method [66]. Health care costs and costs of informal care were calculated as the multiplication of reported utilization and costs per unit in Euros for 2019 [67–69]. The costs of the DICE intervention performed in daily practice (excluding research protocol costs) was calculated based on invested time, personnel cost, and overhead. For the cost-effectiveness calculation, it was assumed that the DICE intervention will be applied once during the first year of patient follow up. The uncertainty for costs, QALYs, and well-being years was assessed by means of non-parametric bootstrapping (5000 observations).

### Ethics

This study was approved by the Medical Ethics Committee of the Erasmus Medical Center in the Netherlands (MEC-2018-1443). Written informed consent was obtained from all participants before study inclusion.

## RESULTS

### Recruitment

**Fig. 2:**
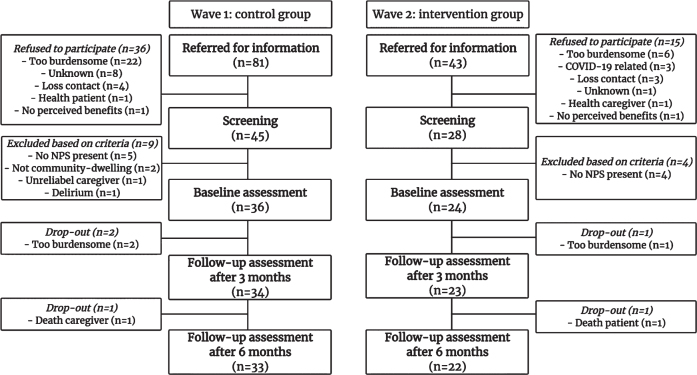
Flow chart of included participants.

The procedure of recruitment is depicted in Fig. 2. After additional screening, 36 patients and their caregivers were included in the control group (44% of the referred patients), while 24 patients were included in the intervention group together with their caregivers (56% of the referred patients). Collaborating physicians reported that a high workload, which was partly due to additional involvement in COVID-19 care, made it hard to refer patients for the intervention group.

### Participants

We included 36 participants in the control group and 24 participants in the intervention group resulting in a total of 60 participants. The majority of participants had AD dementia (77%), while 11 participants had MCI (18%) and three participants (5%) were diagnosed with mixed AD/VaD dementia. Participants were enrolled shortly following diagnosis (median 1.6 months). Of the individuals with dementia, the majority had mild dementia (mean [SD] MMSE score = 23.0 [3.8], 90% CDR score ≤1). Cerebrospinal fluid analysis or amyloid-β PET scan were conducted in 18 participants (30%) and indicated an AD-like biomarker profile in accordance with the clinical diagnosis. One patient was a known APP-mutation carrier. The majority of the patients were born and raised in the Netherlands (93%), while four patients (7%) had a diverse background (*n* = 2 Suriname, *n* = 1 Indonesia, *n* = 1 Germany). All but two caregivers (*n* = 1 Netherlands Antilles, *n* = 1 Germany) were born in the Netherlands and three caregivers (5%) were descendant of a first-generation immigrant. At baseline, we found no differences in demographic and clinical characteristics between the two groups (Table 1).

**Table 1:** Clinical and demographic characteristics at baseline according to group

	Control group	Intervention group
	(*n* = 36)	(*n* = 24)
Department included, N (%)		
Neurology	23 (63.9%)	15 (62.5%)
Geriatrics	13 (36.1%)	9 (37.5%)
*Characteristics patients*		
Age, mean (SD)	73.1 (7.7)	72.5 (6.9)
Female, N (%)	16 (44.4%)	12 (50.0%)
Education, median (IQR)^a^	4.5 (1.0)	5.0 (1.0)
Clinical diagnosis, N (%)		
MCI	9 (25.0%)	2 (8.3%)
AD dementia	24 (66.7%)	22 (91.7%)
Mixed AD dementia/VaD	3 (8.3%)	0 (0.0%)
Months after diagnosis, median (IQR)	1.6 (3.6)	1.6 (1.5)
CDR score			
0.5 (very mild)	17 (47.2%)	7 (29.2%)
1 (mild)	16 (44.4%)	15 (62.5%)
≥2 (moderate to severe)	3 (8.3%)	2 (8.3%)
AD-biomarker signature, N (%)^b^	9 (25.0%)	9 (37.5%)
MMSE score, mean (SD)	23.8 (3.8)	23.5 (3.9)
NPI-Q total score, median (IQR)^c^	14.0 (15.0)	11.5 (26.0)
No. NPS on NPI-Q, median (IQR)^c^	5.0 (4.0)	3.5 (5.0)
Cognitive enhancers, N (%)	19 (47.8%)	11 (45.8%)
Cholinesterase inhibitor	17 (42.2%)	11 (45.8%)
Memantine	2 (5.6%)	0 (0.0%)
Psychotropic drugs, N (%)	6 (16.7%)	5 (20.8%)
Antidepressant	5 (13.9%)	5 (20.8%)
Sedative-hypnotic	1 (2.8%)	1 (4.2%)
Antipsychotic	0 (0.0%)	0 (0.0%)
Mood stabilizer	0 (0.0%)	0 (0.0%)
*Characteristics caregivers*		
Age, mean (SD)	65.9 (11.0)	64.9 (13.0)
Female, N (%)	26 (72.2%)	14 (58.3%)
Education, median (IQR)^a^	5.0 (1.0)	5.0 (1.0)
Relationship to patient, N (%)		
Spouse or partner	28 (77.8%)	19 (79.2%)
Child	8 (22.2%)	5 (20.8%)
Lives together with patient, N (%)	27 (75.0%)	19 (79.2%)

AD, Alzheimer’s disease; CDR, Clinical Dementia Rating scale; MCI, mild cognitive impairment; MMSE, Mini-Mental State Examination; NPI-Q, Neuropsychiatric Inventory-Questionnaire; NPS, neuropsychiatric symptoms; VaD, vascular dementia. ^a^Dutch education system categorized into (1) less than 6 years primary education [<6 years], (2) completed primary education [6 years], (3) more than 6 years of primary education, without a secondary school diploma [8 years], (4) lower vocational training [9 years], (5) advanced vocational training or lower professional education [10–11 years], (6) advanced professional training or upper secondary school [12–18 years], and (7) academic degree [>18 years]. ^b^Established based on either cerebrospinal fluid analysis (amyloid-β_42_ <550 pf/mL or tau/amyloid-β_42_ ratio >0.52) or visual inspection of an amyloid-β PET scan. ^c^missing score for *n* = 1. ^*^p < 0.05 difference between control group and intervention group based on analysis of variance, Mann-Whitney tests, or *χ*^2^ tests.

Three participants (8%) dropped out of the control group because two caregivers experienced participating as too burdensome, and one caregiver deceased during the study leading to a nursing home admission of the patient. Two participants (8%) dropped out the intervention group as one caregiver experienced participating as too burdensome and one patient deceased. We found no substantial differences in baseline characteristics between participants who dropped out of the study and those who completed the study (Supplementary Table 2).

### Quantitative outcomes

#### Primary outcomes

**Fig. 3:**
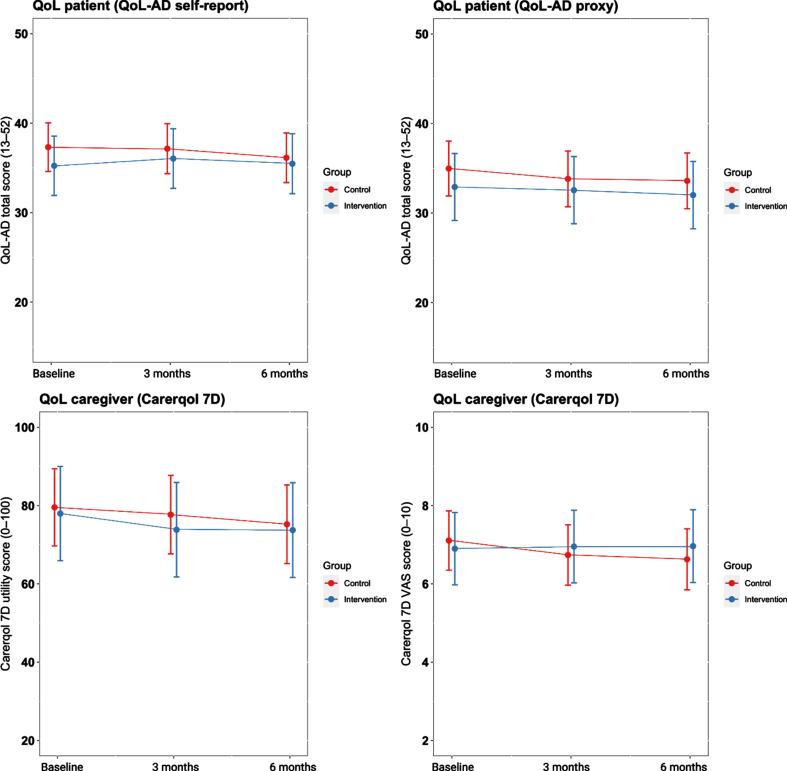
Primary outcomes over time according to group. QoL, quality of life.

We found no effect of the intervention compared to care as usual on changes in self-reported QoL-AD scores (β= 0.20, *p* = 0.37) and proxy QoL-AD scores (β= 0.14, *p* = 0.43) over three months follow-up (Fig. 3). Furthermore, the intervention group did not differ in trajectories of CarerQol-7D utility scores (β= –0.12, *p* = 0.54) and CarerQol-7D VAS scores (β= 0.30, *p* = 0.16) over three months compared to the control group. Effects did not change after six months (all *p* > 0.05) (Table 2).

**Table 2:** Primary and secondary outcomes for the intervention group compared to the control group after three and six months follow-up

	3 months follow-up		6 months follow-up	
Measure	Standardized estimate	*p*	Standardized estimate	*p*
	[95% CI]		[95% CI]	
QoL-AD self-report	0.20 [–0.23, 0.62]	0.37	0.28 [–0.15, 0.71]	0.20
QoL-AD proxy	0.14 [–0.21, 0.50]	0.43	0.08 [–0.28, 0.44]	0.65
Carerqol 7D utility score	–0.12 [–0.51, 0.26]	0.54	0.01 [–0.39, 0.40]	0.98
Carerqol 7D VAS scale	0.30 [–0.11, 0.72]	0.16	0.39 [–0.04, 0.81]	0.08
Perseverance time^a^	2.12 [0.13, 34.23]	0.60	3.10 [0.20, 48.13]	0.42
NPI-Q total score^b^	–0.15 [–0.61, 0.30]	0.51	–0.17 [–0.64, 0.29]	0.46
NPI-Q average distress^b^	0.01 [–0.58, 0.60]	0.96	0.07 [–0.52, 0.66]	0.81
NPI-Q average competence	0.67 [0.02, 1.32]	0.04*	0.34 [–0.32, 1.00]	0.31
BEHAVE-AD total score^b^	–0.09 [–0.45, 0.27]	0.63	0.01 [–0.35, 0.37]	0.97
Psychotropic drug use^b^	0.68 [0.01, 110.61]	0.88	2.64 [0.02, 398.46]	0.71

Estimates are standardized beta coefficients (β) for continuous outcomes and odds ratios (OR) for discrete outcomes derived from linear mixed models corrected for age, sex, disease severity (mild cognitive impairment/dementia), and hospital. BEHAVE-AD, Behavioral Pathology in Alzheimer’s Disease Rating Scale; NPI-Q, Neuropsychiatric Inventory-Questionnaire; QoL-AD, Quality of Life in Alzheimer’s Disease. ^a^Perseverance time categories: >2 years, 1-2 years, <1 year. ^b^Scores were inverted so that positive estimates indicate a positive effect of the intervention (e.g., decrease of NPI-Q total score, NPI-Q average distress, BEHAVE-AD total score, and psychotropic drug use). ^*^p < 0.05, ^**^FDR-corrected *p* < 0.05.

### Secondary outcomes

Compared to the control group, the intervention group showed a significant increase in competence while managing NPS as measured using the NPI-Q over three months follow-up (β= 0.67, *p* = 0.04). This effect did not survive correcting for multiple comparisons (FDR-corrected *p* > 0.05), and diminished after six months (β= 0.34, *p* = 0.31). The intervention did not have an effect on the course of NPI-Q total scores and NPS-related distress (all *p* > 0.05). In addition, there were no differences between the intervention group and the control group in trajectories of perseverance time and psychotropic drug use over six months follow-up (all *p* > 0.05) (Table 2).

We found a significant increase in CMAI total scores (β= 0.41, *p* = 0.01) and RAID total scores in the intervention group compared to the control group after six months (β= 0.82, *p* = 0.02), which did not survive correcting for FDR (all FDR-corrected *p* > 0.05) (Supplementary Table 3). We found no differences between the two groups regarding trajectories of AES-I total scores, CSDD total scores, BEHAVE-AD psychosis scores, SDI total scores, and the presence of specific NPI items (all *p* > 0.05) (Supplementary Table 3).

### Reliable change index

Eleven participants of the intervention group (52%) showed reliable improvement on at least one of the primary outcomes after three months and/or six months follow-up and were therefore classified as ‘responders’ (Table 3). We found no differences between responders and non-responders in demographic characteristics at baseline. Responders showed a higher degree of NPS-related distress as measured using the NPI-Q (median [IQR] = 2.3 [1.0]) compared to non-responders (median [IQR] = 2.0 [1.0], *p* = 0.02). We observed higher NPI-Q total scores at baseline among responders (median [IQR] = 25.0 [21.0]) compared to non-responders (median [IQR] = 9.5 [25.0]), although not statistically significant (*p* = 0.28). Also, responders tended to have a lower disease severity (46% CDR score = 0.5) compared to non-responders (8% CDR score = 0.5), although not statistically significant (*p* = 0.19). A smaller proportion of responders used cognitive enhancers at baseline (27%) compared to non-responders (67%, *p* = 0.04).

**Table 3:** Baseline demographic and clinical characteristics of responders and non-responders in the intervention group

	Responders	Non-responders
	(*n* = 11)	(*n* = 12)
*Characteristics caregivers*		
Age, mean (SD)	68.0 (18.0)	73.0 (21.0)
Female, N (%)	8 (72.7%)	6 (50.0%)
Education, median (IQR)^a^	5.0 (1.0)	5.0 (1.0)
Relationship to patient, N (%)		
Spouse or partner	8 (72.7%)	10 (83.3%)
Child	3 (27.3%)	2 (16.7%)
Lives together with patient, N (%)	8 (72.7%)	10 (83.3)
Perseverance time, N (%)		
>2 year	8 (72.7%)	10 (83.3%)
1-2 years	0 (0.0%)	1 (8.3%)
<1 year	3 (27.3%)	1 (8.3%)
*Characteristics patients*		
Age, median (IQR)	77.0 (10.0)	75.0 (8.0)
Female, N (%)	5 (45.0%)	6 (50.0%)
Education, median (IQR)^a^	5.0 (2.0)	5.0 (1.0)
Clinical diagnosis, N (%)		
MCI	2 (18.2%)	0 (0.0%)
AD dementia	9 (81.8%)	12 (100.0%)
CDR score		
0.5 (very mild)	5 (45.5%)	1 (8.3%)
1 (mild)	5 (45.5%)	10 (83.3%)
≥2 (moderate to severe)	1 (9.1%)	1 (8.3%)
MMSE score, median (IQR)	25.0 (6.0)	23.0 (6.0)
Cognitive enhancers, N (%)^b^	3 (27.3%)	8 (66.7%)*
Psychotropic drugs, N (%)^c^	3 (27.3%)	2 (16.7%)
BEHAVE-AD total score, median (IQR)	5.0 (7.0)	4.5 (7.0)
NPI-Q total score, median (IQR)	25.0 (21.0)	9.5 (25.0)
No. NPS on NPI-Q, median (IQR)	4.0 (4.0)	2.5 (6.0)
NPI-Q average distress, median (IQR)	2.3 (1.0)	2.0 (1.0)*
NPI-Q average competence, median (IQR)	2.0 (0.9)	2.6 (1.4)
NPI-Q delusions, N (%)	3 (27.3%)	3 (25.0%)
NPI-Q hallucinations, N (%)	2 (18.2%)	3 (25.0%)
NPI-Q agitation, N (%)	2 (18.2%	4 (33.3%)
NPI-Q depression, N (%)	9 (81.8%)	8 (66.7%)
NPI-Q anxiety, N (%)	5 (45.5%)	3 (25.0%)
NPI-Q euphoria, N (%)	2 (18.2%)	0 (0.0%)
NPI-Q apathy, N (%)	10 (90.9%)	5 (41.7%)*
NPI-Q disinhibition, N (%)	0 (0.0%)	3 (25.0%)
NPI-Q irritability, N (%)	7 (63.6%)	7 (58.3%)
NPI-Q aberrant motor behavior, N (%)	2 (18.2%)	5 (41.7%)
NPI-Q sleep disturbances, N (%)	4 (36.4%)	1 (8.3%)
NPI-Q eating behavior, N (%)	3 (27.3%)	5 (41.7%)

AD, Alzheimer’s disease; CDR, Clinical Dementia Rating scale; MCI, mild cognitive impairment; MMSE, Mini-Mental State Examination; NPI-Q, Neuropsychiatric Inventory-Questionnaire; NPS, neuropsychiatric symptoms. ^a^Dutch education system categorized into (1) less than 6 years primary education [<6 years], (2) completed primary education [6 years], (3) more than 6 years of primary education, without a secondary school diploma [8 years], (4) lower vocational training [9 years], (5) advanced vocational training or lower professional education [10-11 years], (6) advanced professional training or upper secondary school [12–18 years], and (7) academic degree [>18 years]. ^b^Cholinesterase inhibitors or memantine. ^c^Antidepressants, sedative-hypnotics, antipsychotics or Mood stabilizers. ^*^p < 0.05 difference between responders and non-responders based on Mann-Whitney tests or *χ*^2^ tests.

Responders showed a higher prevalence of apathy (91%) as measured with the NPI-Q at baseline compared to non-responders (42%, *p* = 0.01). Several other NPI-Q domains were endorsed more prevalent among responders compared to non-responders, although not statistically significant, including sleep disturbances (36% versus 8%, *p* = 0.10), anxiety (46% versus 25%, *p* = 0.30), euphoria (18% versus 0%, *p* = 0.12), and depressive symptoms (82% versus 67%, *p* = 0.41). In contrast, non-responders showed higher prevalence compared to responders on NPI-Q domains including disinhibition (25% versus 0%, *p* = 0.08), aberrant motor behavior (42% versus 18%, *p* = 0.22), agitation (33% versus 18%, *p* = 0.41), although not statisticallysignificant.

### Qualitative outcomes

Twelve patients and their caregivers of the intervention group (50.0%) agreed to participate in a semi-structured interview after the last follow-up assessment was completed. Identified themes were: 1) substantial heterogeneity among participants, and 2) experiences with the intervention.

There was considerable heterogeneity among participants regarding the symptoms that caused most distress. While the majority of patients and caregivers reported NPS including apathy, irritability, and/or psychotic symptoms as most distressing, four participants reported solely difficulties due to cognitive problems, such as memory or language deficits. Furthermore, there was substantial variation in the degree of caregiver burden among caregivers. Several participants experienced serious burden while caring for the patient in terms of emotional distress and/or having to assist in daily activities:

“He sees things that are not real and, every morning, I have to assist him with dressing up and showering. It feels like a constant battle. ⋯  Sometimes it’s OK, but we have fights over twenty times a day.” (participant #09, spouse of male with dementia).

However, five spouses did not consider themselves a caregiver:

“I visit a peer support group for dementia caregivers, but actually, I don’t see myself as a caregiver at all. For example, last month, I went on a four-day city trip with a friend of mine, while my husband stayed at home alone, which was absolutely fine for the both of us.” (participant #01, spouse of male with dementia)

In line with this, there were differences among participants to which extent they felt supported by family and friends and had to ask them for help.

A few participants spontaneously mentioned benefits of the intervention. Some of these experiences were related to the management of NPS (e.g., dealing with negative emotions), while other experiences were not specific for NPS (e.g., disclosing the diagnosis to family and friends). One caregiver reported that the intervention was too short, and another caregiver indicated that the intervention would have been more effective if it was delivered sooner because of the extent of cognitive impairment and NPS at this stage. There were no clear differences between responders and non-responders regarding causes of distress, caregiver burden, and the availability of social support.

### Cost-effectiveness

Average health care costs for six months per patient did not significantly differ between the intervention group (€ 2751) and in the control group (€ 2417, *p* = 0.88) (Supplementary Table 4). The non-significant difference observed was very close to the average cost of the DICE intervention (€ 327). After six months, the intervention group did not differ from the control group in the number of QALYs (*p* = 0.72) and well-being years (*p* = 0.75) (Supplementary Table 4). The cost-effectiveness analysis showed that switching from care as usual to the intervention led to an increase in health care costs and societal costs, while QALYs and well-being years remained relatively stable (Supplementary Table 4). This resulted in negative incremental costs per QALY. The probability that the intervention produces more QALYs and well-being years than care as usual ranged between 37–52%, while the probability that the intervention saves health care and societal costs ranged between 45–46%.

## DISCUSSION

Main findings of the present study were that 1) the DICE method did not improve QoL in patients with early AD dementia and their caregivers visiting the memory clinic, 2) there was a trend of increase in the intervention group in confidence managing NPS and severity of agitation and anxiety compared to the control group, and 3) in exploratory analysis, treatment-related benefits in QoL were related to higher levels of baseline NPS-related distress among caregivers, higher baseline prevalence of apathy, and less cognitive deficits at baseline.

We found no effects of the intervention on QoL of patients and caregivers. Positive effects on QoL have rarely been reported for care programs similar to the DICE method, as QoL have rarely been used as outcome measure and studies that did include such measures did not find an effect [70, 71]. Furthermore, baseline QoL measures were high in our sample (Fig. 3), compared to previous European studies among community-dwelling patients with mild AD dementia [72–76]. In addition, QoL measures remained relatively stable over time in the control group. Therefore, there might be little room for improving QoL measures in this sample. Another explanation might be that we enrolled a clinically divers population in the intervention group in terms of NPS presence, NPS severity, and cognitive impairment at baseline (Table 1), which reflects the memory clinic population [1, 77]. The heterogeneity was also further emphasized by the outcomes of the semi-structured interviews. These showed that several caregivers do not experience any NPS-related distress, while others experience major burden due to NPS. Furthermore, some caregivers do not consider themselves as a caregiver, while other caregivers did as they have to assist in a variety of activities of daily living. As this could be expected based on our liberal inclusion criteria, we conducted an RCI analysis to examine whether specific subgroups of participants did benefit from the intervention. These exploratory analyses revealed reliable improvement in QoL among caregivers with high levels of baseline NPS-related distress, and in patients with higher prevalence rates of specific NPS and in the mild stages of AD dementia. The finding that participants in the mild clinical stages of AD benefited most from the DICE method may be due to the interventions provided. Interventions such as psychoeducation were provided to both patients and caregivers, and patients with less cognitive impairments may have benefited more from these interventions compared to patients with severe memory deficits. Furthermore, exploratory RCI analyses suggest that caregivers with high levels of NPS-related distress and patients with specific NPS such as apathy, sleep disturbances, and affective symptoms responded better to the DICE method than individuals with agitation-related NPS. While individuals with early-stage AD present with various NPS at the memory clinic [1], these outcomes may inform clinicians to apply the DICE method in those who report substantial NPS-related distress and exhibit specific NPS such apathy, sleep disturbances, and affective symptoms.

We found a significant improvement of confidence in managing NPS after three months follow-up among caregivers in the intervention group compared to the control group. Although this association was not statistically significant after correcting for multiple testing and diminished after six months follow-up, large effects sizes were found for three months follow-up and six months follow-up (Table 2). An increase in confidence while managing NPS has also been found in two previous studies that evaluated the effectiveness of the DICE method to improve the assessment and management of NPS in caregivers and care professionals [38–40]. In addition, we also found an increase in the severity of agitation and anxiety symptoms after six months follow-up. This might result from an increase of awareness of NPS among caregivers due to the intervention, as caregivers may not have been aware that NPS are an integral part of AD dementia before [78]. Also, the increase in agitation and anxiety observed in the intervention group could result from COVID-19 restrictions as a recent meta-analysis showed an increase in NPS among patients with dementia and MCI during COVID-19 lockdowns [79]. The intervention group was recruited during COVID-19 pandemic and a part of the assessments of the control group (29%) and the intervention group (25%) were conducted during a lockdown. However, comparing assessments during lockdown with those not during a lockdown across groups did not reveal any significant differences in primary and secondary outcomes (all *p* > 0.05).

There were no significant gains in QALYs and well-being years following the intervention resulting in large uncertainties regarding positive or negative effects and additional costs or savings. Health related QoL and well-being was relatively high for the patients in the study, which may be partly due to the inclusion of MCI and mild AD dementia. Due to the small sample size, results should be viewed only as explorative. The small difference in health care costs in the intervention group was almost identical to the costs of the intervention itself suggesting that other health care costs were highly similar for both groups. Optimizing the intervention and repeating this study in a larger sample with a longer follow up might be an option to get better information for physicians, patients, and policy makers on the cost-effectiveness of this intervention.

### Strengths and limitations

Strengths of the current study include addressing the whole spectrum of NPS in AD that represents the memory clinic population and using a combination of quantitative and qualitative outcome measures. However, this study also has some limitations. First, this study was conducted during the COVID-19 pandemic, which has affected the enrollment of participants resulting in a lower number of participants than was anticipated on [42]. Consequently, the power to detect an effect was limited. As standardized estimates indicated large effects for caregiver burden and competence managing NPS, future studies that include larger sample sizes are expected to find significant improvement on the clinical outcomes included. Although lockdowns did not seem to affect our primary and secondary outcomes, COVID-19 related restrictions may have affected benefitting from the DICE method among participants as these restrictions had a major impact on the well-being of both patients and caregivers. Second, this study examined the efficacy of the DICE method in a research setting and can thus be classified as a stage II study (pure “efficacy”) according to the NIH Stage Model for Behavioral Intervention Development [80]. Therefore, future studies are needed that study the implementation of the DICE method in the memory clinic setting. A recent study by our group suggests several challenges that need to be overcome prior to implementing care programs such as the DICE method in the memory clinic [32]. For example, there is currently no consensus among memory clinic physicians on whether the care for NPS in early AD dementia should be located at the memory clinic at all, with a substantial proportion of the Dutch memory clinic physicians arguing that this should primarily be located within primary care instead [32]. Addressing challenges like these seem imperative prior to implementation of the DICE method. Finally, only a third of the included participants had their clinical diagnosis of MCI or AD dementia supported by AD-biomarkers. This may have led to in the inclusion of non-AD pathologies, although patients with substantial vascular pathology and those that met additional criteria for non-AD neurodegenerative diseases were excluded. A priori, we intended sensitivity analyses in patients with positive AD biomarkers [42], this was not possible given the low sample size (*n* = 9 pergroup).

### Conclusion

This study shows no benefits for QoL of the DICE method in individuals who visit the memory clinic with early AD. However, findings do suggest that patients with substantial NPS burden and mild AD dementia and caregivers with high levels of NPS-related distress might benefit from a structured care program addressing NPS, which might contribute to the early assessment and adequate management of NPS in early AD.

## Supplementary Material

Supplementary Material

## Data Availability

The data supporting the findings of this study are available on request from the corresponding author. The data are not publicly available due to privacy or ethical restrictions.
